# Food Traceability System Design Incorporating AI Chatbots: Promoting Consumer Engagement with Prepared Foods

**DOI:** 10.3390/foods14213731

**Published:** 2025-10-30

**Authors:** Bingjie Lu, Decheng Wen, Han Li, Xiao Chen

**Affiliations:** 1 School of Management, Shandong University, Jinan 250100, China; 2 School of Economics & Management, Huzhou University, Huzhou 313000, China

**Keywords:** traceability system design, food traceability system, artificial intelligence, food quality, food packaging, prepared foods, consumer responses, consumer engagement, perception of system ease of use

## Abstract

Industrialized processing has increased the complexity of the food supply chain. Concerns about food-related risks have increased consumer interest in food traceability. Traceability systems are regarded as effective tools for mitigating information asymmetry and enhancing food quality and safety. However, the design of traditional food traceability systems overlooks the risk of information overload. Based on information overload theory, this study designs an artificial intelligence (AI) traceability assistant as an innovative tool to optimize traditional food traceability systems and examines its positive effects. This study focuses on prepared foods as the research objects, selecting three types of prepared foods (Kung Pao chicken, fish-flavored shredded pork, and pickled fish) and three food traceability tasks (preservatives, sweeteners, and drug residues) as experimental stimuli. Through three online scenario experiments, 747 valid responses were collected. This study explores the impact of AI traceability assistant design on positive consumer engagement behaviors and its underlying mechanism. The results reveal that the AI traceability assistant significantly promotes positive consumer engagement behaviors. This positive effect is mediated by perceived system ease of use. Furthermore, perceived product risk positively moderates the impact of the AI traceability assistant on perceived system ease of use. Perceived product risk strengthens the mediating effect of perceived system ease of use. This study contributes a novel theoretical perspective for research on food traceability systems and reveals the underlying mechanism through which the AI traceability assistant exerts its positive effect. In practice, it provides actionable guidance for food producers implementing digital traceability solutions.

## 1. Introduction

Industrialized processing has increased the complexity of the food supply chain [1]. The lack of transparency and the presence of information asymmetry are common issues in the food market [2,3], potentially leading to food fraud [4]. Concerns about food-related risks have increased consumer interest in food traceability [5]. The International Organization for Standardization (ISO) defines traceability as “the ability to follow the movement of a feed or food through specified stage(s) of production, processing, and distribution” in ISO 22005 [6]. Designing traceability systems that provide consumers with more product information is an effective approach to alleviating consumer concerns [2,7] and building consumer trust [8]. Previous research has already confirmed the benefits of traceability systems in reducing information asymmetry [7,9,10] and enhancing food quality and safety [11]. In practice, governments and companies have implemented traceability systems [12]. Adding quick response (QR) codes to food packaging enables consumers to access product traceability information through scanning the QR codes [10].

Although food traceability systems can reduce information asymmetry between consumers and producers, the design of traditional traceability systems still has limitations that require optimization [3]. Firstly, the information retrieval design of traditional traceability systems adopts a “static menu query mode” [13]. When searching for information, users can only rely on themselves to identify relevant content and filter out irrelevant information. As a result, the effectiveness of traditional traceability systems largely depends on consumer participation [1]. Secondly, consumers have diverse preferences for traceability information [14,15]. For example, some are interested in food raw materials, while others focus more on the production and processing stages. Currently, the design of traditional traceability systems cannot provide customized information. Furthermore, information processing abilities vary across individuals [16]. For instance, some consumers can comprehend the information in testing report and make decisions based on it, whereas others may feel confused during the information-processing process. For the latter group, the information provided by the traceability system is unlikely to play a meaningful role. Therefore, researchers emphasize that designing traceability systems that can efficiently convey traceability information that meets consumers’ needs is a critical issue that requires special attention [3].

For consumers, traceability information should be tailored to meet their needs and interests as fully as possible [1]. Information that is easy to process and comprehend tends to be more popular [15]. The development of artificial intelligence (AI) provides technical support for overcoming the limitations of traditional traceability system design. Integrating AI into traceability systems is expected to create more innovative application and synergistic effects [3]. AI chatbots are computer programs that utilizes artificial intelligence technology to interact with users online [17]. Using natural language processing and machine learning techniques, AI chatbots can perform tasks such as analyzing information, providing quick responses, and delivering targeted information to users [17,18]. The emergence of AI chatbots has not only changed how consumers obtain information [19] but also enhanced the shopping experience [20]. The development of information and communication technologies has provided technical support for integrating AI with other systems [18]. Currently, AI chatbots have been widely applied in fields such as retailing, marketing, healthcare, education, and tourism [18,20,21]. However, there are still research gaps in the optimization of traceability system design. Inspired by the application of AI chatbots in other fields [22,23,24], this study designs an AI traceability assistant by integrating AI chatbots into traditional traceability systems. The AI traceability assistant enables users to ask questions in natural language and is responsible for retrieving, filtering, and integrating traceability information, thereby enabling the traceability systems to provide users with targeted and customized traceability information.

This study focuses on prepared foods as the research subject for the following reasons. With the rapid development of urbanization and the acceleration of modern life, prepared foods have attracted widespread attention for their convenience and quick preparation [25,26]. The data show that, in 2024, the market size of prepared foods in China reached 485 billion yuan [27], demonstrating significant market potential. However, the market penetration rate of prepared foods in China remains only 10% to 15% [28], while the number of related companies exceeds 70,000. This indicates that companies in the prepared food industry are facing intense market competition. Prepared foods, as highly processed foods, involve complex production and processing processes. From raw material sourcing to the use of additives and other stages of production and processing, there exists significant information asymmetry between consumers and food producers, making it difficult for consumers to assess food quality and safety. This is one of the main factors hindering the market expansion of the prepared foods. Therefore, it is necessary to design a traceability system for prepared foods that can effectively convey traceability information. In addition, this study further examines the positive effects of the AI traceability assistant design on consumer responses to validate the effectiveness of this design. Positive consumer engagement is a key research focus in the field of consumer behaviors [29,30]. Promoting positive consumer engagement can directly (e.g., increasing food sales and market share) or indirectly (e.g., improving word-of-mouth and promoting the conversion of potential customers) contribute to favorable performance outcomes for food companies [29,31], helping them build sustainable competitive advantages [32]. Therefore, prepared food companies can accelerate market diffusion, increase market share, and establish long-term competitive advantages by promoting positive consumer engagement. Based on this, the study explores the impact of the AI traceability assistant design on positive consumer engagement and its underlying mechanism.

To facilitate a better understanding of the traditional traceability system design, AI traceability assistant design and consumer engagement, a detailed literature review is provided in the Supplementary Materials, which, in addition to the research cited above, incorporates key findings from [33,34,35,36,37,38,39,40,41,42,43,44,45,46,47,48,49,50,51,52,53]. The literature review reveals that there are still research gaps in the field of food traceability system. First, existing studies have primarily explored the benefits of traceability systems based on information asymmetry theory, neglecting the information overload that consumers may experience when faced with traceability information. Second, although some scholars have suggested that applying AI technology in traceability systems may generate positive effects, they did not specify how AI technology could be applied to traceability systems, nor did they conduct empirical validation. Therefore, the contributions of this study are as follows. (1) Based on information overload theory, and combining research on traceability systems and AI chatbots, this study designs an AI traceability assistant as an innovative tool to optimize traditional food traceability systems. (2) It reveals the underlying mechanism through which the AI traceability assistant exerts its positive effect. (3) It provides actionable guidance for food producers implementing digital traceability solutions.

In summary, this study identifies the limitations of traditional food traceability systems and proposes an AI traceability assistant design. Accordingly, this study aims to explore: the impact of the AI traceability assistant design on positive consumer engagement behaviors (H1), the mediating role of perceived system ease of use in the relationship between the AI traceability assistant design and positive consumer engagement behaviors (H2), and the moderating role of perceived product risk (H3). A detailed hypothesis development is provided in the Supplementary Materials, incorporating key insights from [54,55,56,57,58] in addition to the research cited above.

## 2. Materials and Methods

### 2.1. Experimental Design

This study adopts the scenario experimental method and compares two food traceability system designs (Traditional traceability system design vs. AI traceability assistant design). The manipulation of the food traceability system designs is achieved by presenting participants with different experimental stimuli. To ensure objectivity in the selection of experimental stimuli, three types of prepared foods (Kung Pao chicken, fish-flavored shredded pork, and pickled fish) were chosen based on comprehensive recommendations from the JD.com platform. Compared with other e-commerce platforms and supermarkets, JD.com establishes the “prepared foods” category to provide a clearer classification of products, which guarantees both the objectivity and representativeness of product selection in this study. In addition, this study selects three food traceability tasks (preservatives, sweeteners, and drug residues) as experimental stimuli. Consumers are concerned about food additives and drug residues and the selection of these traceability tasks reflects consumer concerns.

This study employs a two-level experimental design. In the control group, the traditional traceability system is designed with reference to Cavite et al. [13], which adopts the static menu query mode. After scanning the QR code on the food packaging, participants enter the first-level page of the traceability system, which displays categories of traceability information (including raw material sources, production, processing, transportation, and storage, among others). Users are required to identify the relevant category of the information they seek and then click to access the second-level page for detailed traceability information. In the experimental group, the traceability system is equipped with an AI traceability assistant. After scanning the QR code on the food packaging, participants enter the traceability system, where they can directly ask questions to the AI traceability assistant. Based on the content of their queries, the AI traceability assistant will generate real-time and tailored traceability information. The design of AI traceability assistant is based on the retail chatbots design proposed by Arce-Urriza et al. [24].

This study conducted three experiments to examine whether the AI traceability assistant design can produce positive effect and to explore its underlying mechanism. Study 1 was conducted to test the main effect of the AI traceability assistant design on positive consumer engagement behaviors (H1). Study 2 modified the stimuli to test the robustness of H1 and further examine the mediating role of perceived system ease of use (H2). Study 3 employed different stimuli to test the robustness of H1 and H2 and explore the moderating role of perceived product risk (H3).

### 2.2. Sampling

First, this study recruited participants through the Credamo platform (https://www.credamo.com), which maintains a large and diverse pool of respondents. Credamo was responsible for distributing the experimental questionnaires to respondents. All participants took part voluntarily, and each received a reward of 1–2 yuan, which helped enhance the objectivity of the sample. Second, to ensure sample diversity and minimize bias, this study employed the platform’s quality control measures. These included IP address restrictions (each IP address could participate only once), geographic distribution controls (to avoid excessive concentration of participants in an area), and non-redundant participation (ensuring that each respondent participated in only one experiment, thereby avoiding practice effect). Additionally, participants were randomly assigned to either the control or experimental group through the platform, which helped prevent selection bias. Finally, to improve sample quality, attention check items were used to filter out invalid responses. This study conducted three experiments, recruiting a total of 800 participants. After excluding invalid responses that failed the attention checks, the final sample consisted of 747 valid participants. Table 1 shows that the sample exhibits a diverse distribution in terms of gender, age, education level, and income.

### 2.3. Measures

The measurement scale for perceived system ease of use was adapted from Davis [44] and Arce-Urriza et al. [24]. The measurement scale for positive consumer engagement behaviors was adapted from Kim et al. [59], Dessart et al. [60], Carlson et al. [61], and Recalde et al. [57]. The scale for perceived product risk was adapted from Yoo et al. [62]. All scales employed a seven-point Likert scale, as detailed in Appendix A (Table A1). This study conducted reliability tests for these scales. Study 1 used the consumer engagement behaviors scale (Cronbach’s alpha = 0.899). Study 2 used the perceived system ease of use scale (Cronbach’s alpha = 0.870) and consumer engagement behaviors scale (Cronbach’s alpha = 0.895). Study 3 used the perceived system ease of use scale (Cronbach’s alpha = 0.818), consumer engagement behaviors scale (Cronbach’s alpha = 0.904) and perceived product risk scale (Cronbach’s alpha = 0.958). The Cronbach’s alpha values of all scales are greater than 0.8, indicating that the scales used in this study have good reliability.

### 2.4. Statistical Analysis

First, this study employs a one-way analysis of variance (ANOVA) to test whether there is a significant difference in the impact of traditional traceability system design and AI traceability assistant design on positive consumer engagement behaviors. One-way ANOVA is a statistical method used to determine whether the means of a continuous dependent variable differ significantly across the levels of a categorical independent variable. A statistically significant difference is considered when the *p*-value is below the 0.05 threshold. In this study, the independent variable is the design of food traceability system, which is a categorical variable with two levels (traditional traceability system design vs. AI traceability assistant design). The dependent variable is positive consumer engagement behaviors, which is a continuous variable. Therefore, one-way ANOVA is an appropriate method for testing whether there is a significant difference in the means of positive consumer engagement behaviors between the two traceability system designs.

Second, this study employed PROCESS v3.3 macro (developed by Andrew F. Hayes) to conduct a bootstrap analysis (5000 resamples, 95% confidence interval). PROCESS is an effective tool for analyzing both mediation and moderation effects. It provides estimates of direct and indirect effects, along with bootstrap confidence intervals. When the 95% confidence interval does not include zero, the effect is considered statistically significant. PROCESS offers multiple models, allowing researchers to select the appropriate model based on the specific research context. In this study, Model 4 was used to examine the mediating role of perceived system ease of use in the relationship between the AI traceability assistant design and positive consumer engagement behaviors. Model 7 was used to test a moderated mediation model, examining whether the indirect effect of the AI traceability assistant design on positive consumer engagement behaviors, through perceived system ease of use, was moderated by perceived product risk.

Statistical software used: IBM SPSS Statistics (version 23; IBM Corp., Armonk, NY, USA).

The stimuli screenshots, attention check items, manipulation check items, randomization procedure and post hoc power analyses are provided in Supplementary Materials.

## 3. Results

### 3.1. Study 1 and Results

A between-subjects experiment was conducted in Study 1 to examine the main effect of the AI traceability assistant design on positive consumer engagement behaviors (H1). A total of 200 participants were recruited through the Credamo platform and randomly assigned to either the control group (Traditional traceability system design) or the experimental group (AI traceability assistant design).

**Figure 1:**
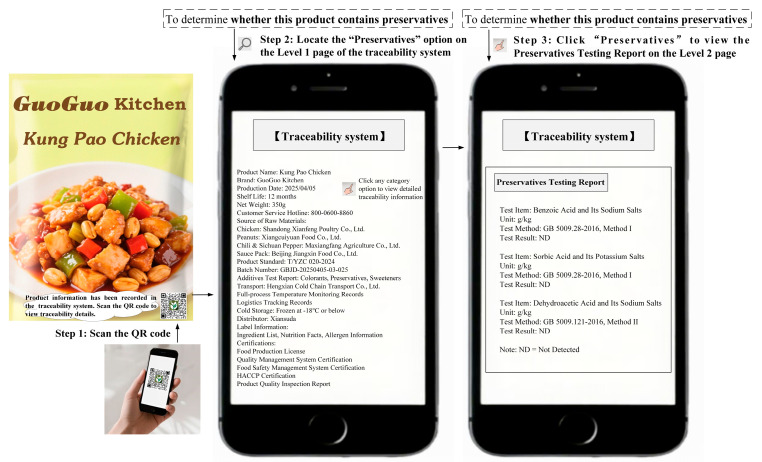
The design of the traditional traceability system (Study 1) [63,64].

**Figure 2:**
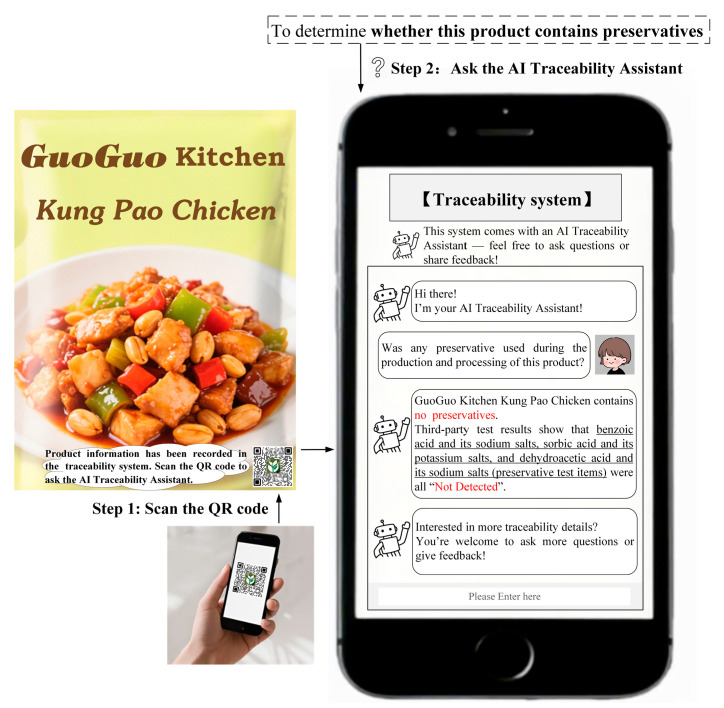
The design of the AI traceability assistant (Study 1).

Study 1 selected Kung Pao chicken as the experimental stimulus and preservatives as the traceability task. Following the experimental method of Treiblmaier and Garaus [3], participants were provided with text and images illustrating the process of using the traceability system to obtain traceability information. The detailed experimental procedure of Study 1 is provided in Appendix B.1. The procedures for obtaining traceability information through the traditional traceability system or AI traceability assistant are shown in Figure 1 and Figure 2, respectively. All participants were required to complete the measurement scale for positive consumer engagement behaviors, the manipulation check, and a set of demographic questions.

#### 3.1.1. Results of Manipulation Test

Study 1 collected 200 questionnaires. After excluding those that failed the attention check, 190 valid responses remained, with 97 in the control group and 93 in the experimental group. A total of 97.37% of participants passed the manipulation check ($\chi^{2}=170.701,\;$p<0.001), indicating that the manipulation of traceability system design was successful.

#### 3.1.2. Results of Main Effect Test

Study 1 employed a one-way ANOVA to test the main effect of the AI traceability assistant design on positive consumer engagement behaviors. To ensure the validity of the ANOVA, normality test and Levene’s test for homogeneity of variance were conducted. The normality test indicated that the data distribution was not significantly skewed. Furthermore, given the sample size of the study, the data can be assumed to approximate a normal distribution, meeting the assumption of normality required for ANOVA. The Levene’s test was not significant ($F\left(1,188\right)=1.663,\;p=0.199>0.05$), indicating that the assumption of homogeneity of variance was satisfied. Therefore, the data were appropriate for ANOVA. The results of the ANOVA are $M_{AI}=5.232,SD_{AI}=0.664;M_{TTS}=4.582,SD_{TTS}=0.739;F(1,188)=40.530,p<0.001,\eta^{2}=0.177$. The results show that in the experimental group (AI traceability assistant design), the mean of positive consumer engagement behaviors is higher than that in the control group (traditional traceability system design). Therefore, compared to the traditional traceability system, the AI traceability assistant significantly promotes positive consumer engagement behaviors, supporting H1.

### 3.2. Study 2 and Results

**Figure 3:**
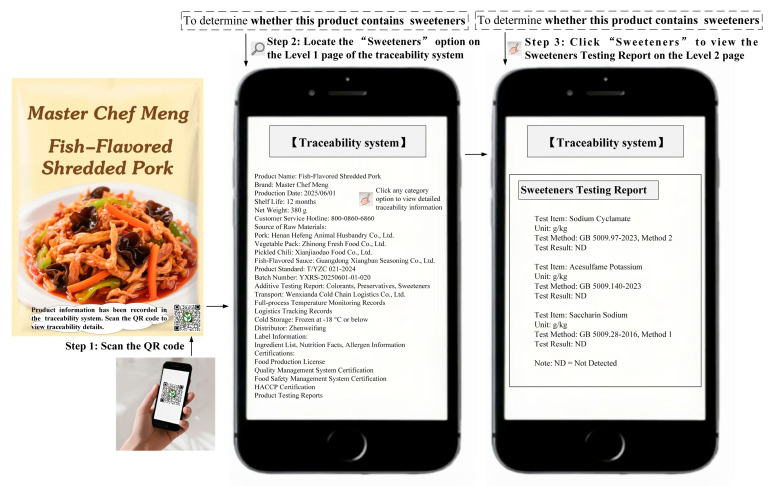
The design of the traditional traceability system (Study 2) [63,65,66].

**Figure 4:**
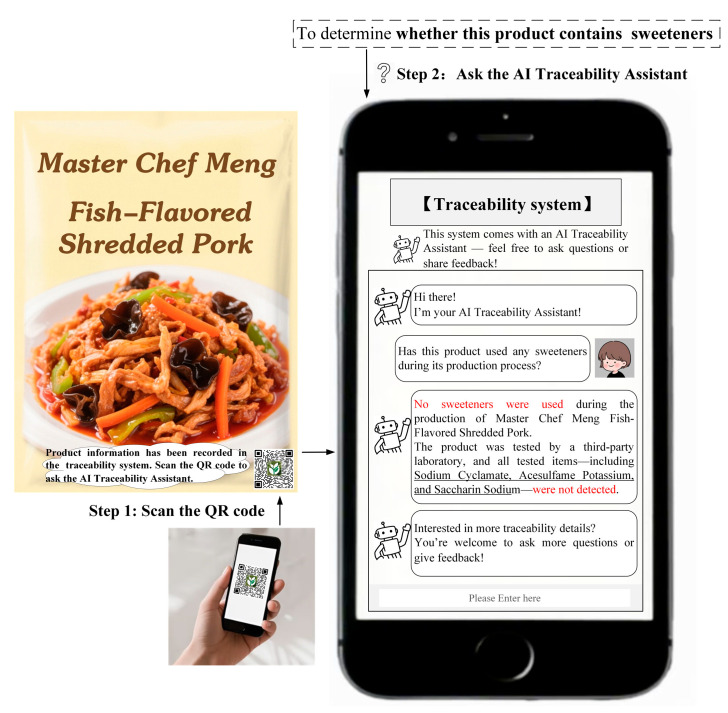
The design of the AI traceability assistant (Study 2).

To test the robustness of H1 and further explore the mechanism through which the AI traceability assistant design influences positive consumer engagement behaviors (H2), Study 2 selected fish-flavored shredded pork as the experimental stimulus and sweeteners as the traceability task. The experimental method was the same as in Study 1, and the detailed procedure is provided in Appendix B.2. The procedures for obtaining traceability information through the traditional traceability system or AI traceability assistant are shown in Figure 3 and Figure 4, respectively. Additionally, food consumption experience, food familiarity, and food preference were included as control variables and measured in the experiment. A total of 200 participants were recruited and randomly assigned to either the control group or the experimental group.

#### 3.2.1. Results of Manipulation Test

Study 2 collected 200 questionnaires. After excluding those that failed the attention check, 188 valid responses remained, with 97 in the control group and 91 in the experimental group. Among the valid responses, 96.81% of participants passed the manipulation check ($\chi^{2}=165.462,p<0.001$), indicating that the manipulation of traceability system design was successful.

#### 3.2.2. Results of Main Effect Test

Study 2 employed a one-way ANOVA to test the robustness of H1. To ensure the validity of the ANOVA, normality test and Levene’s test were conducted. The results indicated that the sample data approximated a normal distribution and met the assumption of homogeneity of variance ($F\left(1,186\right)=1.251,\;p=0.265>0.05$). Therefore, the data were appropriate for ANOVA. The results of the ANOVA are $M_{AI}=5.220,SD_{AI}=0.683;M_{TTS}=$$4.780,SD_{TTS}=0.746;F(1,186)=17.710,p<0.001,\eta^{2}=0.087$. The results show that in the experimental group (AI traceability assistant design), the mean of positive consumer engagement behaviors is higher than that in the control group (traditional traceability system design). Therefore, compared to the traditional traceability system, the AI traceability assistant significantly promotes positive consumer engagement behaviors. This finding further confirms the robustness of H1.

#### 3.2.3. Results of Mediation Effect Test

To further explore the mechanism through which the AI traceability assistant design influences positive consumer engagement behaviors, Study 2 employed PROCESS Model 4 to test the mediation effect of perceived system ease of use (5000 resamples, 95% confidence interval). Demographic variables (gender, age, education, and income), consumption experience, food familiarity, and food preference were included as control variables. When the 95% confidence interval does not include zero, the mediation effect is considered statistically significant The results show that when perceived system ease of use serves as a mediator, the total effect of the AI traceability assistant design on positive consumer engagement behaviors is significant (effect=0.516,BootSE=0.109,95%CI=[0.161,0.590]), the mediating effect of perceived system ease of use is significant (effect=0.284,BootSE=0.110,95%CI=[0.067,0.497]), while the direct effect of the AI traceability assistant design on positive consumer engagement behaviors is no longer significant (effect=0.232,BootSE=0.117,95%CI=[−0.061,0.394]). These results indicate that the AI traceability assistant promotes positive consumer engagement behaviors by enhancing perceived system ease of use, thereby supporting H2. The results of the mediation model are presented in Table 2, and PROCESS results are shown in Table 3.

### 3.3. Study 3 and Results

**Figure 5:**
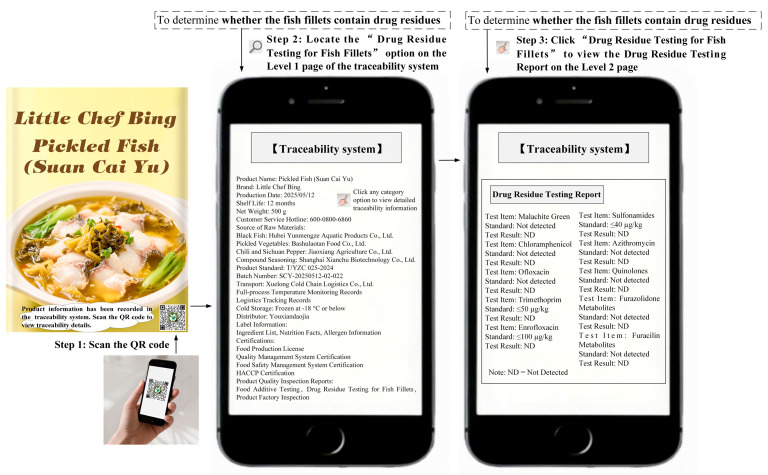
The design of the traditional traceability system (Study 3).

**Figure 6:**
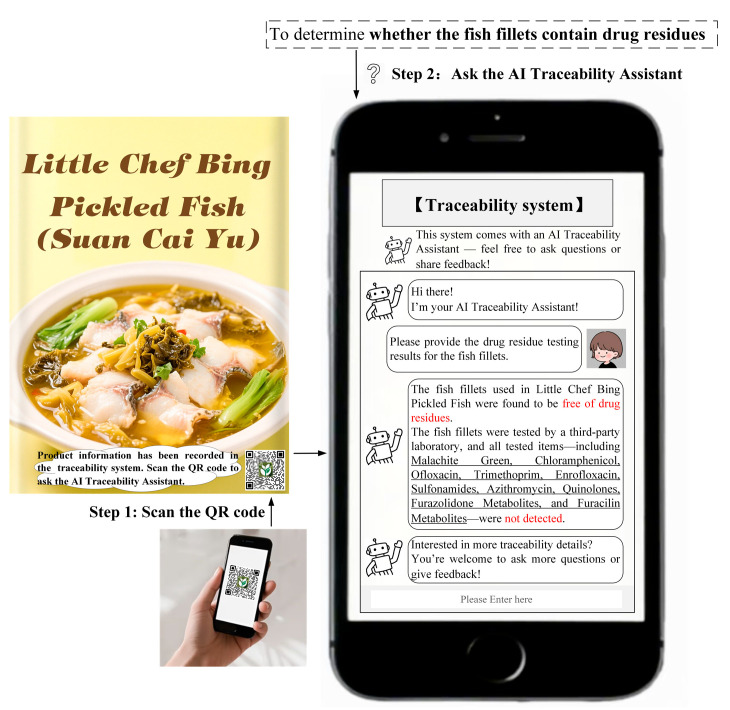
The design of the AI traceability assistant (Study 3).

To test the robustness of H1 and H2 and further explore the moderating effect of perceived product risk, Study 3 selected pickled fish (Suan Cai Yu) as the experimental stimulus and drug residues as the traceability task. This study conducted a 2 (Traditional traceability system design vs. AI traceability assistant design) × 2 (Perceived product risk: low vs. high) between-subjects experiment. The experimental manipulation of the traceability system design was the same as in Study 1, and the manipulation of perceived product risk was adapted from the experimental design of Mollenkopf et al. [67]. The detailed experimental procedure of Study 3 is provided in Appendix B.3. The procedures for obtaining traceability information through the traditional traceability system or AI traceability assistant are shown in Figure 5 and Figure 6, respectively. A total of 400 participants were recruited, and randomly assigned to one of the four groups.

#### 3.3.1. Results of Manipulation Test

Study 3 collected 400 questionnaires, of which 369 valid responses remained after excluding those that failed the attention check. Specifically, with 92 in group 1 (Traditional traceability system design × low), 93 in group 2 (AI traceability assistant design × low), 93 in group 3 (Traditional traceability system design × high), and 91 in group 4 (AI traceability assistant design × high). A total of 94.58% of participants passed the manipulation check of the traceability system design ($\chi^{2}=295.574,p<0.001$). Moreover, the independent sample t-test of perceived product risk was significant ($M_{High}=6.105,M_{Low}=2.796;t=32.592,p<0.001$). The results show that the manipulation was successful.

#### 3.3.2. Results of Main Effect Test

Study 3 employed a one-way ANOVA to further verify the robustness of H1. To ensure the validity of the ANOVA, a normality test and Levene’s test were conducted, and the results indicated that the data met the assumptions for ANOVA ($F\left(1,367\right)=0.396,\;$p=0.529>0.05). The results of the ANOVA are $M_{AI}=5.194,SD_{AI}=0.793;M_{TTS}=4.818,SD_{TTS}$$=0.847;F(1,367)=19.449,p<0.001,\eta^{2}=0.050$. The results show that in the experimental group (AI traceability assistant design), the mean of positive consumer engagement behaviors is higher than that in the control group (traditional traceability system design). Therefore, compared to the traditional traceability system, the AI traceability assistant significantly promotes positive consumer engagement behaviors, thereby providing further support for the robustness of H1.

#### 3.3.3. Results of Moderated Mediation Effect Test

**Figure 7:**
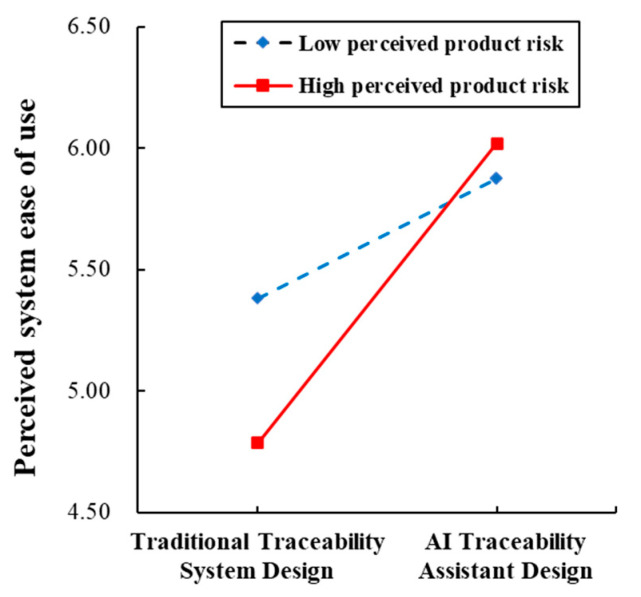
Moderation effect.

To examine the moderating role of perceived product risk, study 3 employed PROCESS Model 7 to conduct a bootstrap analysis (5000 resamples, 95% confidence interval). Demographic variables (gender, age, education, and income), consumption experience, food familiarity, and food preference were included as control variables. As shown in Table 4, compared with the traditional traceability system, the AI traceability assistant significantly improves perceived system ease of use (coeff=0.863,p<0.001), and this effect is stronger under conditions of high perceived product risk (see Figure 7). This indicates that perceived product risk amplifies the positive effect of the AI traceability assistant design on perceived system ease of use (coeff=0.739,p<0.001).

Furthermore, the results in Table 5 show that the mediation effect of perceived system ease of use is statistically significant under both low and high perceived product risk ($effect_{Low}=0.142,BootSE=0.038,95\%CI=[0.076,0.222];effect_{High}=0.354,BootSE=0.072,95\%CI=$[0.216,0.501]), confirming the robustness of H2. It indicates that the AI traceability assistant promotes positive consumer engagement behaviors by enhancing perceived system ease of use. Moreover, the mediation effect of perceived system ease of use is stronger under conditions of high perceived product risk ($effect_{High-Low}=0.212,BootSE=0.055,95\%CI$=[0.112,0.329]), showing that perceived product risk strengthens the mediating role of perceived system ease of use. Therefore, H3 is supported. This indicates that as perceived product risk increases, the AI traceability assistant has a stronger positive effect on perceived system ease of use, which in turn more significantly promotes positive consumer engagement behaviors.

## 4. Discussion

Traceability systems provide many benefits, including reducing information asymmetry, improving food quality and safety, and building consumer trust. Although the design of traditional traceability system can also provide consumers with additional product information, it overlooks the risk of information overload. Drawing on research on traceability systems and AI chatbots, this study designs an AI traceability assistant as an innovative tool to optimize traditional traceability systems. To demonstrate the positive effects of this design, the study further examines its impact on consumer engagement and the underlying mechanism. The following are the findings of this study.

Firstly, Study 1 finds that, compared to the traditional traceability system, the AI traceability assistant design significantly promotes positive consumer engagement behaviors. Existing research has confirmed the positive impact of AI chatbots on consumer behaviors in various fields. For instance, Kumar et al. [18] found that AI chatbots can enhance consumer experience. Wang et al. [23] showed that employing AI livestream assistant can increase revenue and reduce product return rates. In the research on traceability systems, Treiblmaier & Garaus [3] argued that the application of AI technology may generate positive effects, they did not specify how AI technology could be applied to traceability systems, nor did they conduct empirical validation. This study has applied AI technology more concretely to the traceability system by designing an AI traceability assistant and has empirically verified its positive effects.

Secondly, Study 2 finds that the AI traceability assistant design enhances perceived system ease of use, which in turn promotes positive consumer engagement behaviors. As proposed by Arce-Urriza et al. [24], AI chatbots enable users to pose queries in natural language and provide them with information services in an interactive way, which can improve perceived ease of use. The findings of Study 2 are consistent with theirs, providing further evidence that AI chatbots can enhance ease of use. Moreover, recent research has increasingly focused on technology-driven consumer engagement. Perceived ease of use is considered one of the antecedents explaining consumer engagement [56]. Consistent with Recalde et al. [57], Study 2 also supports the positive impact of perceived ease of use on positive consumer engagement behaviors. Furthermore, although some scholars have pointed out that the application of AI technology may optimize traceability systems [3], the underlying mechanism has yet to be explained. The findings of Study 2 explain the mechanism from the perspective of the Technology Acceptance Model (TAM).

Thirdly, Study 3 examines the moderating role of perceived product risk, finding that perceived product risk positively moderates the effect of the AI traceability assistant on perceived system ease of use. Furthermore, under high perceived product risk, the mediating effect of perceived system ease of use is stronger. Given fraud in the food market [3], consumers may receive low-quality or even counterfeit products, making it difficult for them to accurately predict whether there are potential losses after purchasing [10]. When consumers perceive high product risk, their demand for product-related information increases [7]. Consumers are often risk-averse [23], and in high-risk situations, they require a more efficient and flexible way to obtain product information. The empirical findings of Study 3 validate that perceived product risk enhances the impact of the AI traceability assistant on perceived system ease of use and reveal that perceived product risk amplifies the mediating effect of perceived ease of use in the relationship between the AI traceability assistant and positive consumer engagement behaviors. In practice, the AI traceability assistant will be more effective for high-risk food categories.

## 5. Conclusions

### 5.1. Theoretical Contributions

In terms of theoretical contributions, this study (1) provides a new theoretical perspective and framework for research on food traceability system; (2) reveals the underlying mechanism through which the AI traceability assistant exerts its positive effect; (3) identifies that perceived product risk is a theoretical boundary condition for the positive role of the AI traceability assistant.

First, previous research on traceability systems has largely been based on information asymmetry theory, positing that traceability systems can reduce information asymmetry between consumers and food producers [3,7,9,10]. These studies have confirmed the benefits of traceability systems in providing more information but overlooked the risk of information overload. Based on information overload theory, this study proposes the use of AI chatbots to optimize traditional traceability systems (designing an AI traceability assistant). This broadens the theoretical perspective of research on traceability systems and provides a theoretical foundation for optimizing the design of traceability systems.

Second, this study not only extends the application of the TAM but also provides a theoretical explanation of how the AI traceability assistant affects positive consumer engagement behaviors. Previous studies have explored the mediating role of perceived value [9], perceived uncertainty [10], perceived product quality [3], and consumer trust [7] in the influence of traceability systems on consumer responses. This study analyzes the mediating role of perceived system ease of use from the perspective of the TAM, thus providing a new framework for understanding the mechanisms through which traceability system affects consumer behaviors.

Thirdly, previous research has pointed out the presence of fraud in the food market [3], which increases consumers’ perceived risk [10]. When consumers perceive a high level of risk associated with a product, they will seek more product information to support their decision-making [7]. This suggests that perceived product risk stimulates consumers’ demand for traceability information. Earlier studies have validated the theoretical boundary role of factors such as brand familiarity [3] and knowledge of traceability systems [2]. This study analyzes the moderating effect of perceived product risk and confirms that it can amplify the mediating effect of perceived system ease of use. The moderated mediation model provides a comprehensive framework for explaining how the AI traceability assistant functions and when it is more effective.

### 5.2. Practical Implications

Firstly, this study finds that the AI traceability assistant significantly enhances the perceived system ease of use, thereby promoting positive consumer engagement behaviors. This finding provides guidance for prepared food companies in designing their traceability systems. When consumers use traditional traceability systems to query information, they must search, filter, and integrate information on their own. For example, when consumers wish to know about the use of preservatives, they must identify the relevant information from various information (such as raw material sourcing, production, and storage). In contrast, the AI traceability assistant design enables consumers to directly ask questions in natural language (e.g., Does this product use preservatives during production?). In this case, consumers do not need to search or filter the information themselves and AI traceability assistant is responsible for filtering and integrating relevant information to provide targeted answers. In addition, consumers can also provide feedback and suggestions through the assistant, improving the consumer experience. Considering that providing traceability information incurs additional costs for companies, this study suggests that companies should prioritize designing a traceability system equipped with AI chatbots to fully leverage the value of traceability information. For companies that have already established traditional traceability systems, they should consider upgrading their systems with AI chatbots to enhance system usability, thereby promoting consumer engagement behaviors.

Secondly, this study finds that the AI traceability assistant design more effectively enhances perceived system ease of use under high perceived product risk. Based on this conclusion, prepared food companies can implement differentiated traceability system designs for different products. Consumers often develop a distrust of similar products if a particular product category has been exposed to quality or safety issues, leading them to perceive higher risks for such products. At the same time, as processed foods, the more complex the processing procedures and the greater the number of stages involved, the higher the degree of information asymmetry between consumers and producers, increasing consumers’ perceived uncertainty and risk. Perceived risk stimulates a strong demand for traceability information. Many food producers have recognized this and developed traceability systems to help consumers obtain additional product information. However, information overload theory notes that providing more information does not necessarily lead to better decision-making support. Therefore, companies need to avoid the risk of overload when addressing information asymmetry. The key to resolving this contradiction lies in designing an effective method for consumers to access traceability information. For food categories that have previously been exposed to quality or safety issues, as well as for highly processed foods, companies should prioritize adopting the AI traceability assistant design to reduce consumers’ difficulties in obtaining and interpreting traceability information, and better meet their information needs, thereby promoting positive consumer engagement behaviors.

Furthermore, although this study confirms the positive effects of the AI traceability assistant, it still faces several challenges in practice. For instance, developing and maintaining a food traceability system requires investment from companies in hardware, software, and personnel. These costs may be burdensome for small-scale businesses. Additionally, data management presents another major challenge. Since the food supply chain spans from farm to table, the traceability system includes data from various stages such as raw materials, production, processing, transportation, and storage. These data may be uploaded by different participants in the supply chain. Ensuring the authenticity and reliability of the data remains an important issue to address.

### 5.3. Limitations and Future Research

Although this study makes several contributions, it still has some limitations that can be improved in future research. Firstly, information asymmetry is a common issue in the food market. This study focused on prepared foods as the research objects and the experiments were conducted in China. Future research could select other types of foods as the research objects and the experiments could be conducted in other countries and regions, thereby further verifying the generalizability of these findings.

Secondly, scholars have pointed out the shortcomings of traditional traceability systems in terms of information reliability and information transmission. Current studies, based on signal theory, have proposed the use of blockchain technology to enhance the information reliability. This study, on the other hand, draws on information overload theory and suggests the application of AI technology to improve the usability of traceability systems. Future research could integrate both information reliability and information transmission into a framework to explore the synergistic effects of blockchain and AI technologies in optimizing the food traceability systems and further analyze their underlying mechanism.

Thirdly, the AI traceability assistant design proposed in this study aims to provide users with more effective and efficient traceability information services. In the future, its role in intelligent diagnosis could be further explored. Specifically, when users query traceability information using the AI assistant, they often provide a variety of unstructured texts (e.g., “The fish-flavored shredded pork I bought is very sweet. Does it contain sweeteners?”). Machine learning can facilitate business intelligence [68]. Drawing on the work of Wang et al. [69], future research could employ systematic text classification techniques and machine learning methods to analyze the textual information generated during user inquiries. This would enable the AI traceability assistant not only to provide traceability information for consumers but also to perform diagnostics, thereby assisting companies in identifying and analyzing potential issues with food products.

Finally, although AI chatbots have been applied in many fields, they have not yet been widely implemented in traceability systems. Based on research on AI chatbots in other fields, this study designs an AI traceability assistant and analyzes its effectiveness. However, it has not yet considered the specific characteristics of AI chatbots. In other fields, many scholars have examined features such as the anthropomorphic characteristics and proactivity of AI chatbots. Therefore, future research could further explore how to motivate consumers to interact with the AI traceability assistant (e.g., through gamification and rewards).

## Figures and Tables

**Table 1:** Demographic Characteristics of Participants.

Demographic Characteristics	Frequency	Percentage
Gender		
Male	536	71.8
Female	211	28.2
Age		
20 years and below	66	8.8
21–30 years	429	57.4
31–40 years	208	27.8
41–50 years	30	4.0
51–60 years	12	1.6
Over 60 years	2	0.3
Education level		
High school/vocational school	29	3.9
Junior college	48	6.4
Undergraduate	524	70.1
Postgraduate	146	19.5
Income		
2000 yuan and below	143	19.1
2001–5000 yuan	155	20.7
5001–10,000 yuan	283	37.9
Above 10,001 yuan	166	22.2

**Table 2:** The results of the mediation model.

	Positive Consumer Engagement Behaviors		Positive Consumer Engagement Behaviors		Perceived System Ease of Use	
	Coeff.	*p*	Coeff.	*p*	Coeff.	*p*
Control variables						
Gender	0.017	0.795	0.002	0.976	−0.059	0.343
Age	0.125	0.103	0.158	0.044	0.128	0.076
Education	−0.059	0.390	−0.081	0.254	−0.082	0.205
Income	0.080	0.306	0.073	0.365	−0.029	0.692
Consumption experience	0.086	0.324	0.051	0.564	−0.135	0.099
Food preference	0.259	0.001	0.297	0.000	0.149	0.031
Food familiarity	−0.024	0.778	0.010	0.907	0.134	0.098
Independent variables						
AI Traceability Assistant Design	0.232	0.141	0.516	0.000	1.105	0.000
Perceived System Ease of Use	0.257	0.001				
R^2^	0.308		0.267		0.381	
F	8.817		8.164		13.754	
	*p* < 0.001		*p* < 0.001		*p* < 0.001	

**Table 3:** The PROCESS results.

	Effect	BootSE	BootLLCI	BootULCI
Total effect	0.516	0.109	0.161	0.590
Direct effect	0.232	0.117	−0.061	0.394
Indirect effect	0.284	0.110	0.067	0.497

**Table 4:** The results of moderation effect test.

	Perceived System Ease of Use	
	Coeff.	*p*
Control variables		
Gender	−0.012	0.882
Age	0.050	0.335
Education	−0.073	0.200
Income	0.012	0.775
Consumption experience	−0.106	0.311
Food preference	0.180	0.000
Food familiarity	−0.002	0.959
Independent variables		
AI Traceability Assistant Design	0.863	0.000
Perceived product risk	−0.228	0.002
AI Traceability Assistant Design × Perceived product risk	0.739	0.000
R^2^	0.409	
F	24.751	
	*p* < 0.001	

**Table 5:** The results of moderated mediation effect.

Mediation Effect	Effect	BootSE	BootLLCI	BootULCI
Low perceived product risk	0.142	0.038	0.076	0.222
High perceived product risk	0.354	0.072	0.216	0.501
Pairwise contrasts	0.212	0.055	0.112	0.329

## Data Availability

The original contributions presented in the study are included in the article. Further inquiries can be directed to the corresponding author.

## Supplementary Materials

The following supporting information can be downloaded at: https://www.mdpi.com/article/10.3390/foods14213731/s1, Figure S1. The experimental stimuli in the control group of Study 1 (Traditional traceability system). Figure S2. The experimental stimuli in the experimental group of Study 1 (AI traceability assistant). Figure S3. The experimental stimuli in the control group of Study 2 (Traditional traceability system). Figure S4. The experimental stimuli in the experimental group of Study 2 (AI traceability assistant). Figure S5. The experimental stimuli in the control group of Study 3 (Traditional traceability system). Figure S6. The experimental stimuli in the experimental group of Study 3 (AI traceability assistant). Table S1. The exclusion counts per study. Table S2. Parameter settings and power analysis results. References [2,3,7,9,10,13,14,15,16,17,18,19,21,22,23,24,29,30,31,32,33,34,35,36,37,38,39,40,41,42,43,44,45,46,47,48,49,50,51,52,53,54,55,56,57,58] are cited in the supplementary materials.

## Appendix A

**Table A1:** Variables and items.

Variables	Items	References
Perceived system ease of use	The food traceability system provides information in a flexible manner	[24,44]
	It is easy to obtain traceability information using this food traceability system	
	Using this food traceability system to access traceability information does not require much mental effort	
	This food traceability system is easy to use	
Perceived product risk	Purchasing this kind of product involves risk	[62]
	Purchasing this kind of product may involve potential losses	
	The decision to purchase this kind of product is risky	
Positive consumer engagement behaviors	I will purchase this product	[57,59,60,61]
	I will repurchase this product	
	I will purchase other products from this brand	
	I share my product usage experience in social interactions	
	I recommend this product to others in social interactions	
	I help others resolve product-related issues in social interactions	
	I share my product usage experience on online platforms	
	I post positive reviews and recommend this product on online platforms	
	I share product-related knowledge on online platforms to help others	
	I proactively provide feedback on my product usage experience to the company	
	I proactively offer constructive suggestions regarding the product and services	
	I proactively provide feedback on my needs for new products	

## Appendix B

### Appendix B.1

Participants in both the control and experimental groups were asked to imagine the following scenario. While purchasing Kung Pao chicken, you wish to obtain the product’s traceability information (e.g., raw material sources, production and processing, transportation and storage). Then, you scan the QR code located at the bottom-right corner of the product packaging with your mobile phone and access the traceability system. To control for extraneous variables, both groups were assigned the same query task “to determine whether this product contains preservatives”.

Next, participants in the control group were instructed, with the aid of text and images, to imagine the following scenario. When seeking to determine whether the product contains preservatives, you must locate the “Preservatives” option on the traceability system’s first-level page. After clicking on “Preservatives,” you enter the second-level page to view the preservative testing report, where you can only rely on yourself to understand the items in the preservative testing report and to interpret the results.

In the experimental group, participants, also guided by text and images, were asked to imagine the following scenario. When seeking to determine whether the product contains preservatives, you can directly ask the AI traceability assistant, “Was any preservative used during the production and processing of this product?” The AI traceability assistant then integrates the relevant information available in the traceability system and provides a direct response.

### Appendix B.2

In Study 2, the product was replaced with fish-flavored shredded pork, and the query task was changed to “determine whether this product contains sweeteners”. The process by which participants in the control and experimental groups accessed traceability information through the traceability system was similar to that in Study 1.

### Appendix B.3

Study 3 employed a 2 (Traditional traceability system design vs. AI traceability assistant design) × 2 (Perceived product risk: low vs. high) between-subjects design. At the beginning of the experiment, participants were presented with a news article concerning drug residue testing in aquatic products, which was used to manipulate the perceived product risk. Subsequently, as in Studies 1 and 2, participants were shown the process of accessing traceability information through the traceability system. In Study 3, the stimulus product was pickled fish (Suan Cai Yu), and the query task was changed to “determine whether the fish filets contain drug residues”. The process by which participants in the control and experimental groups accessed traceability information through the traceability system was similar to that in Study 1.

## References

[1] Rodriguez-Salvador B., Dopico D.C. (2020). Understanding the value of traceability of fishery products from a consumer perspective. Food Control.

[2] Rao S., Chen F., Hu W., Gao F., Huang J., Yi H. (2023). Consumers’ valuations of tea traceability and certification: Evidence from a blockchain knowledge experiment in six megacities of China. Food Control.

[3] Treiblmaier H., Garaus M. (2023). Using blockchain to signal quality in the food supply chain: The impact on consumer purchase intentions and the moderating effect of brand familiarity. Int. J. Inf. Manag..

[4] Guo C., Liu Y., Na M., Song J. (2023). Dual-Layer Index for Efficient Traceability Query of Food Supply Chain Based on Blockchain. Foods.

[5] Shahzad K., Zhang Q., Zafar A.U., Ashfaq M., Rehman S.U. (2023). The role of blockchain-enabled traceability, task technology fit, and user self-efficacy in mobile food delivery applications. J. Retail. Consum. Serv..

[6] (2007). Traceability in the Feed and Food Chain—General Principles and Basic Requirements for System Design and Implementation.

[7] Lam T., Heales J., Hartley N. (2024). The Impacts of Traceability Systems on Consumer Trust. J. Comput. Inf. Syst..

[8] Hoque M.Z., Akhter N., Chowdhury M.S.R. (2022). Consumers’ Preferences for the Traceability Information of Seafood Safety. Foods.

[9] Yuan C., Wang S., Yu X. (2020). The impact of food traceability system on consumer perceived value and purchase intention in China. Ind. Manage. Data Syst..

[10] Wu X., Xiong J., Yan J., Wang Y. (2021). Perceived quality of traceability information and its effect on purchase intention towards organic food. J. Market. Manag..

[11] Kechagias E.P., Gayialis S.P., Papadopoulos G.A., Papoutsis G. (2023). An Ethereum-Based Distributed Application for Enhancing Food Supply Chain Traceability. Foods.

[12] Liu R., Wang J., Liang J., Ma H., Liang F. (2023). Perceived Value of Information Attributes: Accounting for Consumer Heterogeneous Preference and Valuation for Traceable Agri-Food. Foods.

[13] Cavite H.J., Mankeb P., Suwanmaneepong S. (2022). Community enterprise consumers’ intention to purchase organic rice in Thailand: The moderating role of product traceability knowledge. Br. Food J..

[14] Jin S., Zhou L. (2014). Consumer interest in information provided by food traceability systems in Japan. Food Qual. Prefer..

[15] Jin S., Zhang Y., Xu Y. (2017). Amount of information and the willingness of consumers to pay for food traceability in China. Food Control.

[16] Chen Y.C., Shang R.A., Kao C.Y. (2009). The effects of information overload on consumers’ subjective state towards buying decision in the internet shopping environment. Electron. Commer. Res. Appl..

[17] Kim J.H., Kim J., Kim C., Kim S. (2023). Do you trust ChatGPTs? Effects of the ethical and quality issues of generative AI on travel decisions. J. Travel Tour. Mark..

[18] Kumar A., Shankar A., Behl A., Chakraborty D., Gundala R.R. (2025). Anthropomorphic generative AI chatbots for enhancing customer engagement, experience and recommendation. J. Consum. Mark..

[19] Foroughi B., Naghmeh-Abbaspour B., Wen J., Ghobakhloo M., Al-Emran M., Al-Sharafi M.A. (2025). Determinants of generative AI in promoting green purchasing behavior: A hybrid partial least squares-artificial neural network approach. Bus. Strateg. Environ..

[20] Hsu C.L., Lin J.C.C. (2023). Understanding the user satisfaction and loyalty of customer service chatbots. J. Retail. Consum. Serv..

[21] Lee K.W., Li C.Y. (2023). It is not merely a chat: Transforming chatbot affordances into dual identification and loyalty. J. Retail. Consum. Serv..

[22] Ferraro C., Demsar V., Sands S., Restrepo M., Campbell C. (2024). The paradoxes of generative AI-enabled customer service: A guide for managers. Bus. Horiz..

[23] Wang L., Huang N., He Y., Liu D., Guo X., Sun Y., Chen G. (2025). Artificial Intelligence (AI) Assistant in Online Shopping: A Randomized Field Experiment on a Livestream Selling Platform. Inf. Syst. Res..

[24] Arce-Urriza M., Chocarro R., Cortiñas M., Marcos-Matás G. (2025). From familiarity to acceptance: The impact of Generative Artificial Intelligence on consumer adoption of retail chatbots. J. Retail. Consum. Serv..

[25] Wooldridge K., Riley M.D., Hendrie G.A. (2021). Growth of Ready Meals in Australian Supermarkets: Nutrient Composition, Price and Serving Size. Foods.

[26] Wumaierjiang R., Xu Y., Wang L., Guo T., Chen G., Li R. (2024). A Cross-Sectional Study of Pre-Prepared Foods Knowledge, Attitudes, and Practices of College Students in Central China. Nutrients.

[27] Blue Book on the Development of China’s Prefabricated Vegetable Industry from 2024 to 2025. https://report.iimedia.cn/repo147-0/57829.html.

[28] Report on the Development of the Prepared Food Industry. http://yjy.people.com.cn/n1/2023/0710/c440911-40031856.html.

[29] Kumar V., Aksoy L., Donkers B., Venkatesan R., Wiesel T., Tillmanns S. (2010). Undervalued or overvalued customers: Capturing total customer engagement value. J. Serv. Res..

[30] Kabadayi E.T., Aksoy N.C., Turkay P.B. (2023). How does customer engagement value occur in restaurants? A stimulus-organism-response (SOR) perspective. Serv. Ind. J..

[31] Hao F. (2020). The landscape of customer engagement in hospitality and tourism: A systematic review. Int. J. Contemp. Hosp. Manag..

[32] Yen C.H., Teng H.Y., Tzeng J.C. (2020). Innovativeness and customer value co-creation behaviors: Mediating role of customer engagement. Int. J. Hosp. Manag..

[33] Vuoso V., Mondelli A., Ceniti C., Venuti I., Ciardella G., Proroga Y.T.R., Nisci B., Ambrosio R.L., Anastasio A. (2025). Assessing Risks and Innovating Traceability in Campania’s Illegal Mussel Sale: A One Health Perspective. Foods.

[34] Wang E.S.T., Tsai M.C. (2019). Effects of the perception of traceable fresh food safety and nutrition on perceived health benefits, affective commitment, and repurchase intention. Food Qual. Prefer..

[35] Nunes L.C., Deliberador L.R. (2025). What motivates people to purchase food products with traceability systems? A structural equation modeling approach. Food Qual. Prefer..

[36] Ahearne M., Atefi Y., Lam S.K., Pourmasoudi M. (2022). The future of buyer–seller interactions: A conceptual framework and research agenda. J. Acad. Mark. Sci..

[37] Duong C., Sung B., Lee S., Easton J. (2022). Assessing Australian consumer preferences for fresh pork meat attributes: A best-worst approach on 46 attributes. Meat Sci..

[38] Jacoby J. (1977). Information load and decision quality: Some contested issues. J. Mark. Res..

[39] Bawden D., Holtham C., Courtney N. (1999). Perspectives on information overload. Aslib Proc..

[40] Wang X., Zhang Z., Jiang Q. (2024). The effectiveness of human vs. AI voice-over in short video advertisements: A cognitive load theory perspective. J. Retail. Consum. Serv..

[41] Eppler M.J., Mengis J. (2004). The concept of information overload: A review of literature from organization science, accounting, marketing, MIS, and related disciplines. Inf. Soc..

[42] Nelson M.R. (1994). We have the information you want, but getting it will cost you! held hostage by information overload. XRDS.

[43] Yao Q., Tao X., Zhou W. (2022). To make your mouth water or not? How field dependence/independence and occasion-setting cues affect consumers’ food intake intention. Eur. J. Market..

[44] Davis F.D. (1989). Perceived usefulness, perceived ease of use, and user acceptance of information technology. MIS Q..

[45] Wistedt U. (2024). Consumer purchase intention toward POI-retailers in cross-border E-commerce: An integration of technology acceptance model and commitment-trust theory. J. Retail. Consum. Serv..

[46] Hellali W., Korai B. (2023). Understanding consumer’s acceptability of the technology behind upcycled foods: An application of the technology acceptance model. Food Qual. Prefer..

[47] Youssef Y.M.A., Johnston W.J., AbdelHamid T.A., Dakrory M.I., Seddick M.G.S. (2018). A customer engagement framework for a B2B context. J. Bus. Ind. Mark..

[48] Kunz W., Aksoy L., Bart Y., Heinonen K., Kabadayi S., Ordenes F.V., Sigala M., Diaz D., Theodoulidis B. (2017). Customer engagement in a big data world. J. Serv. Mark..

[49] Do D.K.X., Rahman K., Robinson L.J. (2020). Determinants of negative customer engagement behaviours. J. Serv. Mark..

[50] Żyminkowska K., Perek-Białas J., Humenny G. (2023). The effect of product category on customer motivation for customer engagement behaviour. Int. J. Consum. Stud..

[51] So K.K.F., Wei W., Martin D. (2021). Understanding customer engagement and social media activities in tourism: A latent profile analysis and cross-validation. J. Bus. Res..

[52] Naumann K., Bowden J., Gabbott M. (2020). Expanding customer engagement: The role of negative engagement, dual valences and contexts. Eur. J. Market..

[53] Do D.K.X., Bowden J.L.H. (2023). Negative customer engagement behaviour in a service context. Serv. Ind. J..

[54] Cui Y.G., van Esch P., Phelan S. (2024). How to build a competitive advantage for your brand using generative AI. Bus. Horiz..

[55] Shahzad M.F., Xu S., An X., Javed I. (2024). Assessing the impact of AI-chatbot service quality on user e-brand loyalty through chatbot user trust, experience and electronic word of mouth. J. Retail. Consum. Serv..

[56] Hollebeek L.D., Belk R. (2021). Consumers’ technology-facilitated brand engagement and wellbeing: Positivist TAM/PERMA-vs. Consumer Culture Theory perspectives. Int. J. Res. Mark..

[57] Recalde D., Jai T.C., Jones R.P. (2024). I can find the right product with AR! The mediation effects of shopper engagement on intent to purchase beauty products. J. Retail. Consum. Serv..

[58] Quevedo-Silva F., Lucchese-Cheung T., Spers E.E., Alves F.V., de Almeida R.G. (2022). The effect of COVID-19 on the purchase intention of certified beef in Brazil. Food Control.

[59] Kim J.W., Choi J., Qualls W., Han K. (2008). It takes a marketplace community to raise brand commitment: The role of online communities. J. Market. Manag..

[60] Dessart L., Veloutsou C., Morgan-Thomas A. (2016). Capturing consumer engagement: Duality, dimensionality and measurement. J. Market. Manag..

[61] Carlson J., Rahman M.M., Taylor A., Voola R. (2019). Feel the VIBE: Examining value-in-the-brand-page-experience and its impact on satisfaction and customer engagement behaviours in mobile social media. J. Retail. Consum. Serv..

[62] Yoo C.W., Parameswaran S., Kishore R. (2015). Knowing about your food from the farm to the table: Using information systems that reduce information asymmetry and health risks in retail contexts. Inf. Manag..

[63] (2016). Determination of Benzoic Acid, Sorbic Acid and Saccharin Sodium in Foods.

[64] (2016). Determination of Dehydroacetic Acid in Foods.

[65] (2023). Determination of Sodium Cyclamate in Foods.

[66] (2023). Determination of Acesulfame Potassium in Foods.

[67] Mollenkopf D.A., Peinkofer S.T., Chu Y. (2022). Supply chain transparency: Consumer reactions to incongruent signals. J. Oper. Manag..

[68] Khan W.A., Chung S.H., Awan M.U., Wen X. (2020). Machine learning facilitated business intelligence (Part I) Neural networks learning algorithms and applications. Ind. Manag. Data Syst..

[69] Wang S., Moon S., Eum I., Hwang D., Kim J. (2025). A text dataset of fire door defects for pre-delivery inspections of apartments during the construction stage. Data Brief.

