# Supplementary material for: Food Traceability System Design Incorporating AI Chatbots: Promoting Consumer Engagement with Prepared Foods
Source: Foods. 2025 Oct 30;14(21):3731. doi: 10.3390/foods14213731 (PMC12609340; doi:10.3390/foods14213731)
Supplement: Supplementary file 1 [file foods-14-03731-s001.zip › foods-3929636-supplementary.pdf]

Supplementary Materials

1. Literature Review

1.1. Application and Limitations of Traditional Traceability Systems

The uncertainty in the production process makes it difficult for consumers to determine food quality and safety [33]. Consumer demand for pre-consumption information has driven the development and application of traceability systems [34]. Supported by Internet of Things (IoT) technologies, traceability systems can track and record product information across production, processing, and distribution stages [13,35]. Companies can use technologies such as quick response (QR) codes to share more product information with consumers, overcoming the limitations of traditional paper labels on food packaging [9,14] and reducing information symmetry between buyers and sellers [36]. Previous research, based on the information asymmetry theory, has explored the benefits of traceability systems, finding that they can enhance perceived value [9], reduce perceived uncertainty [10] and strengthen consumer trust [7]. These studies suggest that traceability systems assist consumers in product evaluation and decision-making by providing additional information [10]. However, the design of traditional traceability systems has not taken into account the information overload that consumers may experience when faced with large amounts of information [37].

The information overload theory suggests that humans have a limited capacity to process information [38]. When this capacity is exceeded, information overload occurs [38]. Information overload is described as a state in which the efficiency of information use is hindered by the volume of available information [39]. Overload will lead to confusion and inaccuracies in decision-making [38,39]. Cognitive psychology presents a similar theory. Cognitive load theory states that human cognitive resources are limited [40]. The more input information there is, the greater the effort required to process it. Once processing capacity is exceeded, overload occurs [16]. For example, when consumers check preservative testing report, the technical terms and various data in the report may lead to information overload and cognitive overload. Although traceability systems provide consumers with information across all stages of production, processing, and distribution [3], consumers tend to focus on and use information that is useful for their judgments and decisions [37]. Moreover, different consumers have varying preferences for traceability information [15] and differing capacities for information processing [16]. Previous research on traceability system has not taken these aspects into account.

The benefits of implementing traceability systems in the food market have been confirmed [9,10]. However, providing traceability information to consumers can be costly [15]. Therefore, it is essential to consider how food traceability systems can be designed to maximize the value of traceability information. The information overload theory provides direction for optimizing the design of traditional traceability systems.

1.2. Food Traceability System Design Incorporating AI Chatbots

The fundamental mechanism to combat information overload is to ensure that the information provided holds high value for the recipient [41]. AI is considered an effective tool for addressing information overload, as it can analyze the relationship between available information and user interests, intelligently filter, compress, and aggregate information, and return personalized results to the user [42]. AI chatbots are a key application of AI technology, using natural language processing and other techniques to simulate human conversational interactions on internet platforms or mobile applications [17]. AI chatbots can understand user

inputs, analyze semantics, and generate contextually relevant responses, providing users with interactive information service [24]. AI chatbots can recognize the information needs of different users and provide targeted and personalized content, thereby catering to diverse information preferences [23]. The development of information and communication technologies has provided the technical support for integrating AI with other systems [18]. The incorporation of AI chatbots into traditional traceability systems provides users with a high-quality intelligent information filter.

The design of traditional traceability systems can only deliver unidirectional information (from the system to the user), and the format and content of traceability information are fixed rather than customized [13]. This may lead to information overload, making it difficult for ordinary consumers to quickly locate the product information they care about. For example, when consumers want to seek information about preservatives in traditional traceability systems, they must rely on themselves to identify relevant information from various information (such as raw material sourcing, production, and storage). Additionally, product information often includes numerous non-experiential cues, such as technical parameters [43]. Traditional traceability systems can only present this information to consumers without offering targeted and personalized explanations. For consumers, such information may be difficult to comprehend. And, traditional traceability systems are unable to provide users with multi-turn guidance. In contrast, AI chatbots provide more interactive information (with bidirectional communication between the system and the user), and the information content is more personalized through providing targeted responses based on user queries [23,24]. In this case, the AI traceability assistant enables consumers to directly ask questions in natural language (e.g., Did this product use preservatives during production?). So, consumers do not need to search or filter the information themselves and AI traceability assistant is responsible for filtering and integrating relevant information to provide targeted and easily understandable response. In addition, consumers can also provide feedback and suggestions through multi-turn conversations with the AI traceability assistant. Therefore, compared to the traditional traceability system design, the AI traceability assistant design offers additional value in terms of interactivity, explainability, and multi-turn guidance , which helps reduce information overload.

Furthermore, the AI traceability assistant proposed in this study differs from the blockchain-based traceability systems discussed in previous research. Blockchain technology ensures that traceability information is unforgeable and tamper-resistant [2]. Previous studies have analyzed the value of blockchain-based traceability system design from the perspective of signal theory, finding that providing more reliable traceability information enhances consumers' perceived product quality [3]. This implies that the innovation of blockchain-based traceability systems lies in using blockchain technology to enhance the reliability of traceability information. However, how to effectively communicate this information to consumers to meet their informational needs remains insufficiently explored [3]. This study, grounded in information overload theory and research on AI chatbots, proposes an AI traceability assistant design. Its innovation lies in using AI technology to optimize the communication of traceability information, addressing the issue which has not been fully tackled by traditional traceability systems or blockchain-based traceability systems.

Arce-Urriza et al. [24] argue that AI chatbots enhance the ease of use due to their advanced natural language understanding capabilities. Ease of use is one of the core variables in the Technology Acceptance Model (TAM), which was initially developed to explain and predict user's acceptance of new technologies [44]. TAM posits that "perceived usefulness" and "perceived ease of use" are the underlying mechanisms through which external variables influence user's behavior [45]. Currently, the scope of TAM has expanded beyond its original

application in information technology to various other fields, and it can also be used to analyze the mechanisms through which certain factors influence consumer responses [46]. For instance, Rao et al. [2] found that perceived usefulness in the TAM framework can explain why individuals who prioritize quality and safety are willing to pay a higher premium for blockchain-based traceability systems.

Drawing on research related to AI chatbots, this study designs an AI traceability assistant to optimize traditional traceability systems. Based on TAM, this study explores the impact mechanism of the AI traceability assistant design on consumer responses from the perspective of perceived ease of use.

1.3. Consumer Engagement

Consumer engagement occurs in interactions between consumer and products, brands, or companies [47]. Some scholars argue that consumer engagement consists of non-transactional behaviors, such as recommending and sharing, which extend beyond transactional behaviors like purchasing, while others contend that consumer engagement encompasses both transactional and non-transactional behaviors [48-50]. The relevant literature highlights the importance of focusing more on consumer engagement behaviors, as the value of customer is derived from behaviors [51]. Moreover, consumer engagement behaviors should not neglect transactional behaviors while focusing solely on non-transactional behaviors [30]. Consumer engagement behaviors can be either positive or negative. Negative consumer engagement behaviors, such as spreading negative word-of-mouth, collective complaints, and boycotts [52], can significantly damage the reputation of companies and result in the loss [53]. Conversely, positive consumer engagement behaviors, such as purchasing, recommending and providing feedback [29], can generate greater customer value and help companies develop the sustainable competitive advantages [32].

Scholars have confirmed that positive consumer engagement behaviors can generate both direct and indirect contributions to companies [47]. For example, repeat purchases and cross-buying can increase food sales, while positive word-of-mouth and recommendations can persuade potential consumers to convert into regular consumers, thereby expanding the food market share [29,31]. Thus, promoting positive consumer engagement behaviors plays a crucial role in the market diffusion of products, including prepared foods.

2. Hypothesis Development

2.1. The Positive Impact of the AI Traceability Assistant Design

Wang et al. [23] argue that searching for information to support decision-making is time-consuming and effort-intensive, and they suggest that designing an AI assistant can ensure that consumers receive timely information services when they have informational needs. AI assistants, essentially AI chatbots, can provide interactive information services, generating context-aware, more targeted, and natural responses [24]. AI chatbots have transformed the way consumers obtain information [23]. Through interaction, AI chatbots assist consumers in making better product choices and provide post-purchase support [22], thereby enhancing the ability to establish effective and lasting relationships with consumers [21,24]. Existing research indicates that using AI chatbots for customer service can improve service efficiency and enhance consumer experience [54]. AI chatbots can help users better understand information, thereby moving beyond passive consumption and generating a more interactive and dynamic engagement [19]. Currently, the application of AI chatbots has been shown to improve consumer experience [18], increase purchase intention [23], and promote consumer behaviors such as purchasing and recommending [18,19]. AI chatbots play a positive role in enhancing consumer engagement [55].

Based on the above research, this study proposes the following hypothesis.

H1: Compared to traditional traceability systems, the AI traceability assistant design promotes positive consumer engagement behaviors.

2.2. The Mediating Role of Perceived System Ease of Use in AI Traceability Assistant Design

AI chatbots enable consumers to pose questions using natural language [24]. They can summarize and simplify diverse information and data related to the product lifecycle, identify the specific information needs of different consumers, and deliver tailored content [19,23]. By doing so, AI chatbots enhance consumers' efficiency in obtaining and processing information [18,23]. Previous studies have shown that interacting with AI chatbots requires minimal effort, and their advanced natural language processing capabilities can significantly increase the perceived system ease of use [24]. The emergence of technologies such as AI has spurred research on technology-driven consumer engagement [56]. Perceived ease of use is not only a construct for predicting consumer trust, commitment, and purchase intention [45], but also regarded as a critical antecedent of consumer engagement [57]. When consumers perceive a technology as easy to use, the barriers to their use of this technology diminish, thereby facilitating engagement [56]. For instance, Recalde et al. [57] found that consumers' perceived ease of use of AR applications significantly promotes positive consumer engagement behaviors, such as sharing, recommending, and providing feedback.

Based on the above research, this study proposes the following hypothesis.

H2: Compared to traditional traceability systems, the AI traceability assistant design improves perceived system ease of use, thereby promoting positive consumer engagement behaviors.

2.3. The Moderating Role of Perceived Product Risk in AI Traceability Assistant Design

Perceived risk refers to the subjective perception of potential losses [23]. Consumers are typically risk-averse, and when a product involves perceived risks, they tend to seek more information to ensure its safety [7,23]. The higher the perceived product risk, the greater the perceived informativeness of traceability systems [7]. The increase in perceived informativeness may also lead to information overload [16]. As argued by Quevedo-Silva et al. [58], although traceability systems hold potential as marketing tools, their impact on consumer

decision-making may vary, as consumers differ in their ability to use such systems and interpret traceability information. The design of traditional traceability systems adopts a static menu query mode [13], in which users can only rely on themselves to search for information and filter out irrelevant information. Product information often contains numerous non-experiential cues (e.g., technical parameters), and the lager number of information categories and attribute options can increase the difficulty of information search and processing, thereby imposing cognitive burdens on consumers [43]. In contrast, AI chatbots can dynamically generate personalized content according to consumer needs [18], thereby improving the efficiency of both information obtaining and processing [23].

AI chatbots can improve system ease of use [24], thereby promoting consumer engagement [56,57]. Under conditions of high perceived risk, perceive informativeness of traceability systems is lager [7]. Consumers are often required to process this information within a limited time, which places substantial pressure on their cognitive capacity [43]. Therefore, this study proposes that under conditions of high perceived product risk, the mediating effect of perceived system ease of use on the relationship between the AI traceability assistant design and positive consumer engagement behaviors will be stronger.

Based on the above research, this study proposes the following hypothesis.

H3: Under high perceived product risk, the AI traceability assistant design has a stronger positive effect on perceived system ease of use, which in turn more significantly promotes positive consumer engagement behaviors.

3. Supplementary Materials and Methods

3.1. Stimuli Screenshots

This study proposes an AI traceability assistant design and examines its effectiveness through three online scenario experiments. Studies 1, 2, and 3 employed different experimental stimuli, selecting three types of prepared foods (Kung Pao Chicken, Fish-Flavored Shredded Pork, and Pickled Fish) and three food traceability tasks (preservatives, sweeteners, and drug residues) as experimental materials. These studies were conducted in China, so the textual stimuli were presented in Chinese. In the Results section of the main text, we provide English translation versions of the stimulus materials to enhance readability (Figures 1-6). Meanwhile, to maintain the originality and authenticity of the experimental stimuli, the original Chinese versions of the stimulus materials are provided in Figures S1-S6.

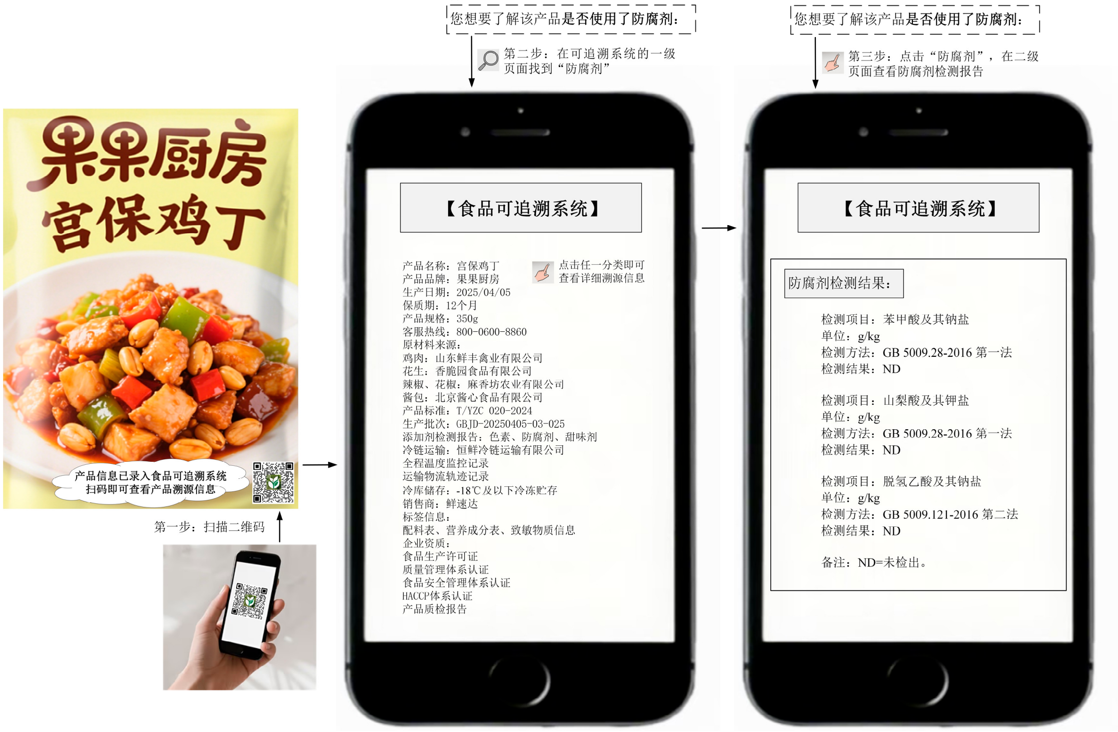

Figure S1. The experimental stimuli in the control group of Study 1 (Traditional traceability system)

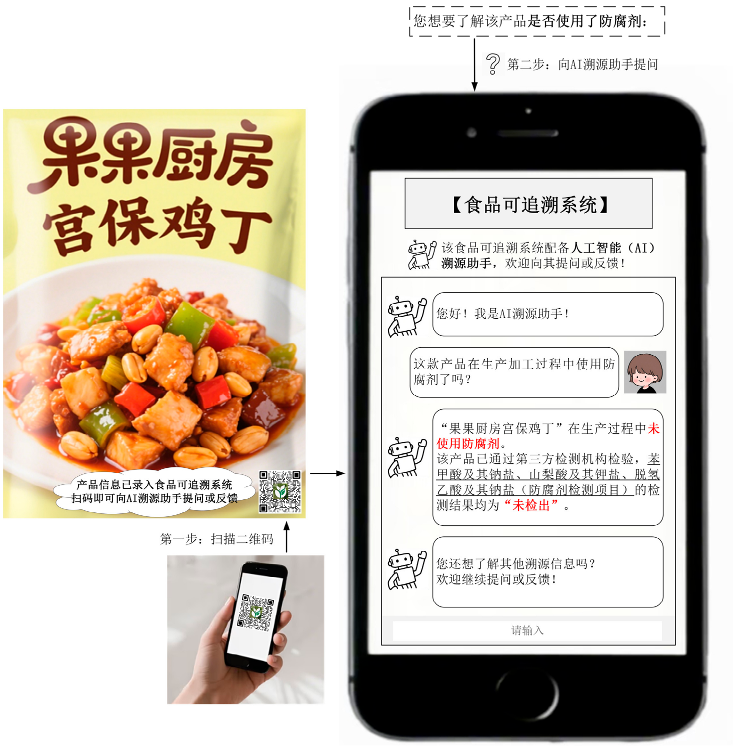

Figure S2. The experimental stimuli in the experimental group of Study 1 (AI traceability assistant)

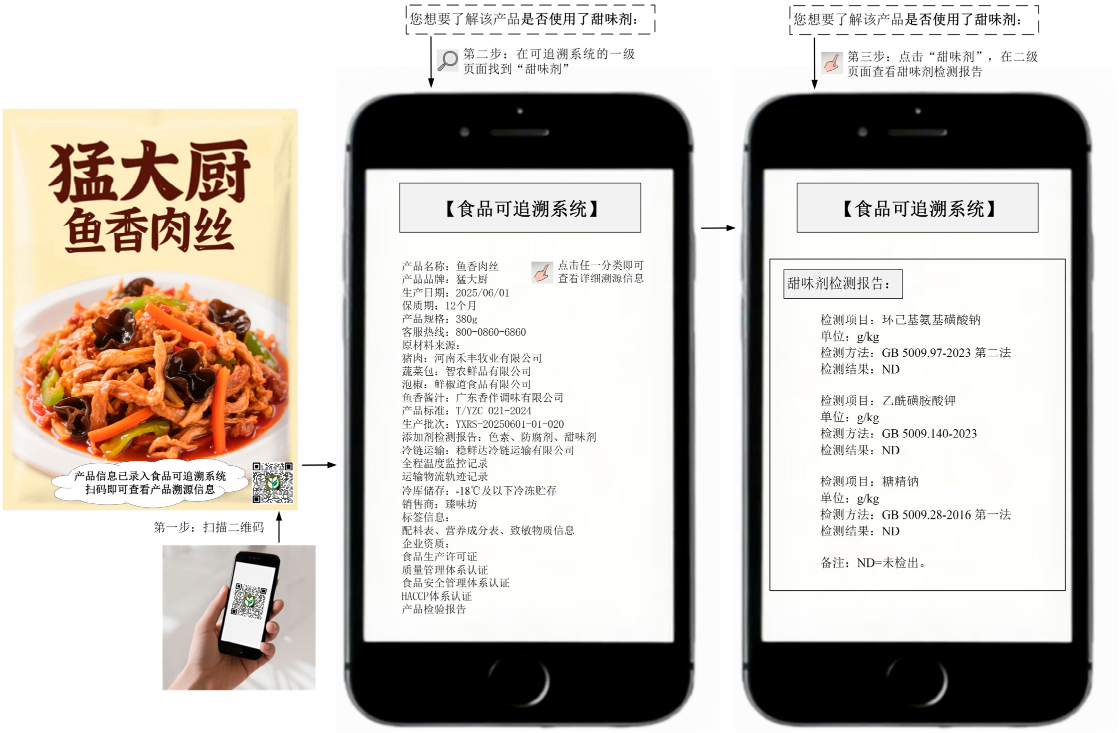

Figure S3. The experimental stimuli in the control group of Study 2 (Traditional traceability system)

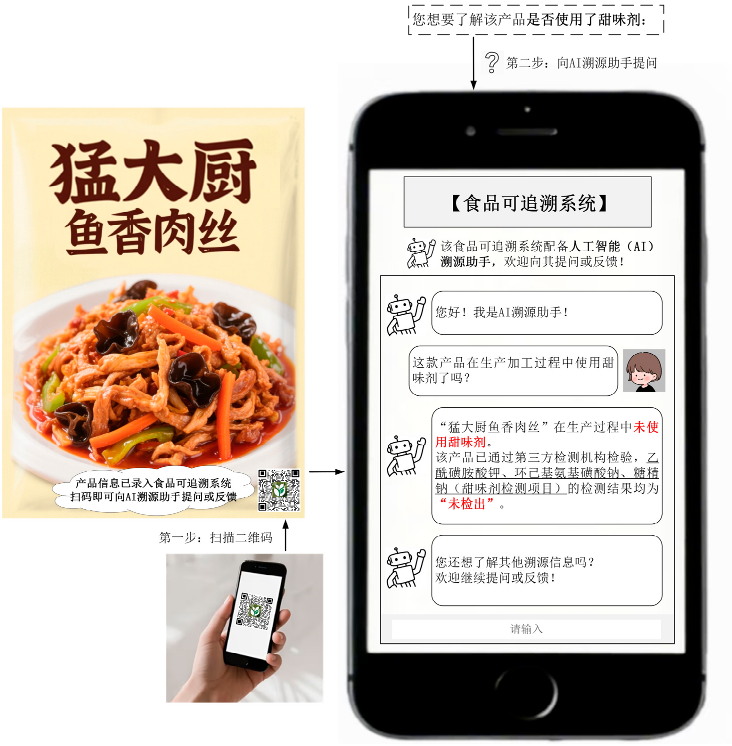

Figure S4. The experimental stimuli in the experimental group of Study 2 (AI traceability assistant)

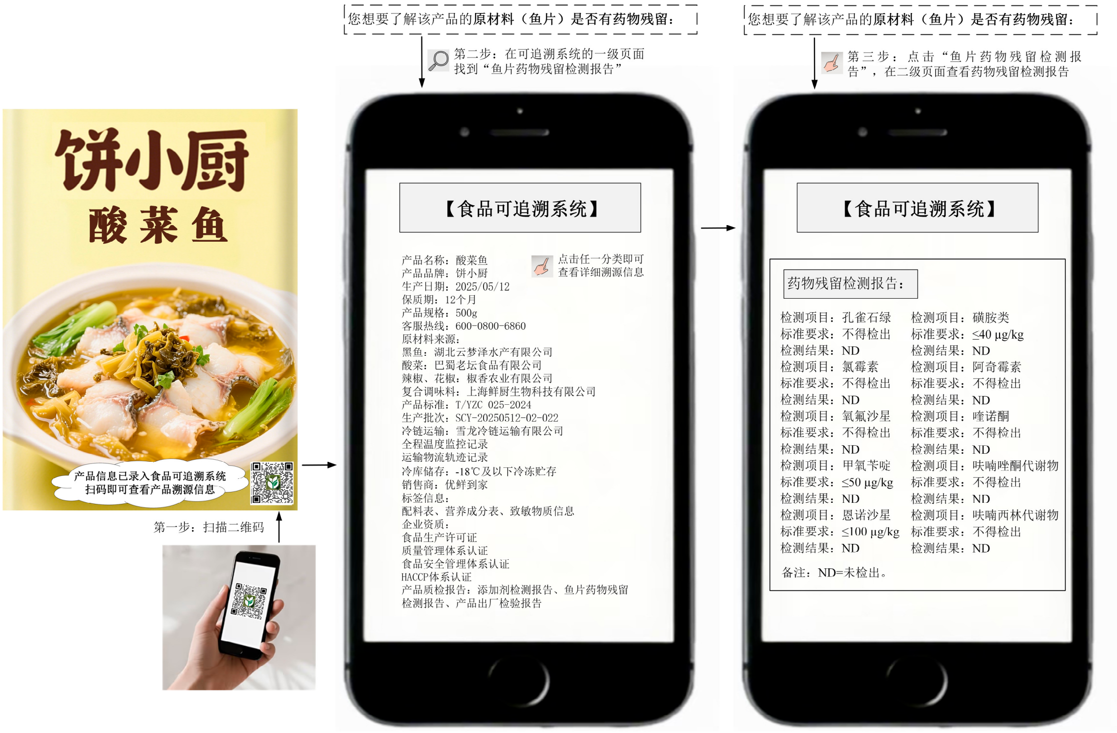

Figure S5. The experimental stimuli in the control group of Study 3 (Traditional traceability system)

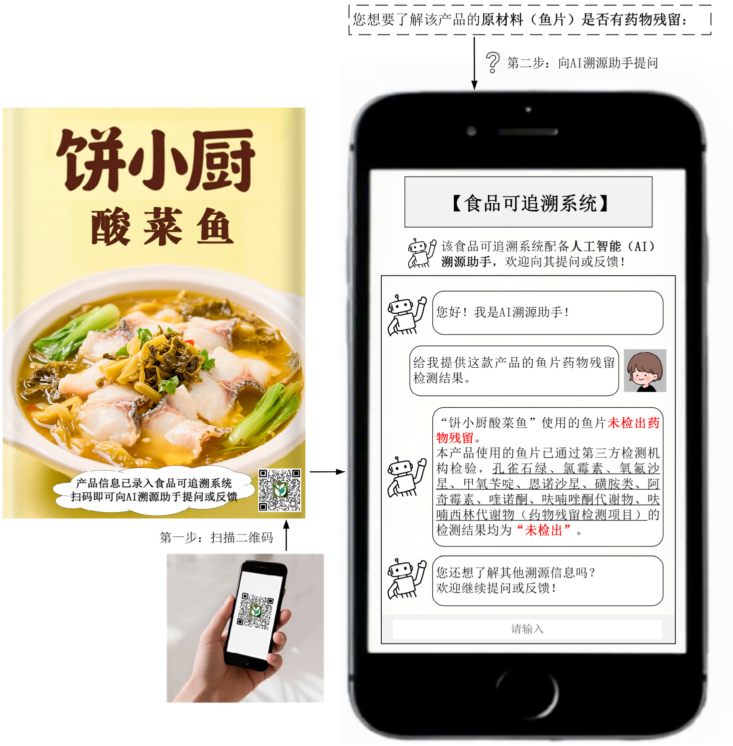

Figure S6. The experimental stimuli in the experimental group of Study 3 (AI traceability assistant)

3.2. Attention Check and Manipulation Check Items

To screen and exclude participants who did not answer attentively, we employed two attention check measures. First, we used the system screening function on the survey platform (Credamo) to assess whether participants answered carefully. The attention check items included "6+3×4=?", "6+3+4=?", and "This question checks whether you are answering attentively, please select 'Very Dissatisfied'." The options for these items were randomized, and participants who answered incorrectly were automatically rejected by the platform and excluded from the final participant count.

Second, to ensure that participants thoroughly read and comprehended the experimental stimuli, we asked them attention check questions related to the stimuli. Specifically, participants were required to rank the steps involved in using the food traceability system to query traceability information. These items were presented after the experimental stimuli to ensure the validity of attention check. The attention check items for the control and experimental groups in Study 1 are shown below.

- Attention check item for the control group (Traditional traceability system)

Select the steps for using the food traceability system to query traceability information (e.g., "To determine whether this product contains preservatives?"):

- ☐ Locate the "Preservatives" on the first page of the food traceability system.
- ☐ Scan the QR code on the product's packaging.
- ☐ Click "Preservatives" to view the preservatives testing report on the second page.
- ☐ Review the testing items and results in the preservatives testing report.

- Attention check item for the experimental group (AI traceability assistant)

Select the steps for using the food traceability system to query traceability information (e.g., "To determine whether this product contains preservatives?"):

- ☐ Ask the AI traceability assistant.
- ☐ Scan the QR code on the product's packaging.

Similar attention check items were used in Studies 2 and 3. In the Results section of the main text, we report the valid sample size for each study. The exclusion counts per study are reported in Table S1.

Table S1. The exclusion counts per study.

Study		Total	Exclusion	Valid
Study 1	Control group (Traditional traceability system)	100	3	97
	Experimental group (AI traceability assistant)	100	7	93
Study 2	Control group (Traditional traceability system)	100	3	97
	Experimental group (AI traceability assistant)	100	9	91
Study 3	Control group (Traditional traceability system)	200	15	185
	Experimental group (AI traceability assistant)	200	16	184
Total		800	53	747

Then, to ensure the validity of the experimental manipulation, each participant was required to answer manipulation check item. The manipulation of the food traceability system design was achieved by presenting different experimental stimuli to the participants. The AI traceability assistant design refers to the integration of an AI Chatbot into the traditional traceability system. Therefore, the difference between the experimental stimuli in the control group and the experimental group lies in the presence or absence of an AI traceability assistant. This manipulation check item was adapted from the study of Treiblmaier & Garaus (2023), which was designed to verify whether participants correctly perceived the scenario they were assigned to. The exact manipulation check item is "Please recall whether the food traceability system in the scenario above included an AI traceability assistant".

3.3. Randomization Procedure

Participants were randomly assigned to different groups using the built-in randomization module of the Credamo platform. The platform automatically and randomly assigned each participant to one of the groups (Traditional traceability system design vs. AI traceability assistant design) with equal probability. This process ensured that the assignment was entirely objective and free from researcher intervention, thereby enhancing the validity of the experimental design. For example, in Study 1, we used the platform's process settings function to set up two modules: one for the control group (Traditional traceability system design) and one for the experimental group (AI traceability assistant design). The random assignment of experimental scenarios was achieved through the platform's random display function. This ensured that each participant was randomly assigned to one of the scenarios.

3.4. Post-hoc Power Analyses

This study conducted a one-way analysis of variance (ANOVA) to test the main effect. To verify the statistical power of the one-way ANOVA, post-hoc power analyses were performed using G*Power for Studies 1, 2, and 3. The G*Power inputs include α, effect size, and sample

size. Specially, α was typically set at 0.05, with the other parameters (effect size and sample size) based on the Results section. The results showed that the statistical power was greater than 0.80, demonstrating that the sample had adequate statistical power. The parameter settings are provided in Table S2.

Table S2. Parameter settings and power analysis results.

	**Study 1**	**Study 2**	**Study 3**
effect size	0.177	0.087	0.050
sample size	190	188	369
α	0.05	0.05	0.05
Power	>0.80	>0.80	>0.80
